# Body-relative horizontal–vertical anisotropy in human representations of traveled distances

**DOI:** 10.1007/s00221-018-5337-9

**Published:** 2018-07-20

**Authors:** Thomas Hinterecker, Paolo Pretto, Ksander N. de Winkel, Hans-Otto Karnath, Heinrich H. Bülthoff, Tobias Meilinger

**Affiliations:** 1 Max-Planck-Institute for Biological Cybernetics, Max-Planck-Ring 8, 72076 Tübingen, Germany; 2 Graduate Training Centre of Neuroscience, Tübingen University, Tübingen, Germany; 3 Division of Neuropsychology, Center of Neurology, Tübingen University, Tübingen, Germany

**Keywords:** Traveled distances, Horizontal, Vertical, Anisotropy, Body-centered, Motion simulator

## Abstract

A growing number of studies investigated anisotropies in representations of horizontal and vertical spaces. In humans, compelling evidence for such anisotropies exists for representations of multi-floor buildings. In contrast, evidence regarding open spaces is indecisive. Our study aimed at further enhancing the understanding of horizontal and vertical spatial representations in open spaces utilizing a simple traveled distance estimation paradigm. Blindfolded participants were moved along various directions in the sagittal plane. Subsequently, participants passively reproduced the traveled distance from memory. Participants performed this task in an upright and in a 30° backward-pitch orientation. The accuracy of distance estimates in the upright orientation showed a horizontal–vertical anisotropy, with higher accuracy along the horizontal axis compared with the vertical axis. The backward-pitch orientation enabled us to investigate whether this anisotropy was body or earth-centered. The accuracy patterns of the upright condition were positively correlated with the body-relative (not the earth-relative) coordinate mapping of the backward-pitch condition, suggesting a body-centered anisotropy. Overall, this is consistent with findings on motion perception. It suggests that the distance estimation sub-process of path integration is subject to horizontal–vertical anisotropy. Based on the previous studies that showed isotropy in open spaces, we speculate that real physical self-movements or categorical versus isometric encoding are crucial factors for (an)isotropies in spatial representations.

## Introduction

The previous studies on spatial representations focused on the horizontal dimension. However, humans and other animals live and move in a three-dimensional (3D) world. For instance, humans frequently travel on uneven terrain and navigate within multi-level buildings. Because of technical achievements, they sometimes also freely fly or scuba dive in volumetric (or open) space. Recognizing this gap in research, a growing body of neurophysiological and psychological studies emerged to investigate representations of 3D space, including spatial locations, directions, etc. (for reviews, see Finkelstein et al. 2016; Jeffery et al. 2013). A common research question of these studies was whether and how the brain creates accurate representations of horizontal and vertical space.

One of the previously conducted neurophysiological studies investigating horizontal and vertical spatial encoding in rats reported higher neuronal sensitivity for locations and translational movements along the horizontal axis compared with the vertical axis (Hayman et al. 2011). The targeted neurons in this study were the so-called place and grid cells, which are located in the hippocampus and the medial entorhinal cortex, respectively (for a review of these cells, see Moser et al. 2015). Whereas place cells represent and indicate the current location of the animal in space (O’Keefe and Dostrovsky 1971), grid cells seem to represent the Euclidean space in which the animal is moving (Fyhn et al. 2004; Hafting et al. 2005). Grid cells are also assumed to be a distance-measuring unit of the spatial cognitive system (Hayman et al. 2011; Jeffery et al. 2015). The above-mentioned findings of Hayman et al. (2011) led to the hypothesis that 3D spatial representations are being subject to anisotropy, with vertical space being encoded less accurately than horizontal space, rather than being encoded isotropically (i.e., equal accuracy) (Jeffery et al. 2013).

Specifically, Jeffery et al. (2013) postulated the bicoded-map hypothesis. This hypothesis states that egocentric horizontal space is represented more accurately than vertical space. Although this hypothesis is consistent with the findings in rats, one may ask whether internal spatial representations are anisotropic for all species. In fact, this seems not to be the case: Yartsev and Ulanovsky (2013) showed that place cells in flying bats seem to encode space similar along the horizontal and vertical dimensions. A study on pelagic fish reported that these fish possess isotropic representations of the environment (Burt de Perera et al. 2016). These findings suggest that isotropy versus anisotropy of 3D spatial representations depends on the investigated species. Possibly, the natural habits and living environments of an animal influence the ability of its spatial system to represent 3D space isotropically (Finkelstein et al. 2016). Whereas animals that move freely in open spaces (like bats, fish, etc.) are able to represent horizontal and vertical space isotropically, surface dwellers (like rats) seem to lack this ability.

What about humans? Do we represent horizontal and vertical space isotropically, or do we, as surface dwellers, encode space along the horizontal dimension with higher accuracy too? Most research that addressed this question involved multi-floor buildings and findings indicate anisotropic representations. Humans seem to represent horizontal space in such buildings (e.g., rooms on the same floor) more accurately than vertical space (across floors) (Brandt et al. 2015; Büchner et al. 2007; Hölscher et al. 2006; Montello and Pick 1993; Thibault et al. 2013; Zwergal et al. 2016).

In contrast to multi-floor studies, the few studies on (an)isotropic representations of horizontal and vertical open spaces are not that clear-cut. In fact, the current evidence rather promotes isotropic representations than anisotropic ones. First, in a recent fMRI study, participants navigated within a virtual, open-space 3D lattice structure. Results showed similar memory accuracy and hippocampus-activity patterns for horizontal (front–back–left–right) and vertical (up–down) location representations (Kim et al. 2017). Second, equal horizontal versus vertical memory accuracy was also found in a setup in which participants learned locations of objects on a table (horizontal) and on an upright board (vertical) from a single viewpoint but recalled them from varying test orientations within the room (Hinterecker et al. 2018). Contrarily to these isotropy findings, a study in which participants were moved through an open space and were required to point to the origin of travels showed anisotropies that depend on the involved spatial plane (Barnett-Cowan et al. 2012). Another study using a point-to-origin task revealed results indicating that the horizontal (yaw) and the vertical (pitch) planes are encoded in different brain areas (Indovina et al. 2016).

Accordingly, the current picture of anisotropy in human representations of horizontal and vertical space suggests that humans are capable of isotropic encoding in some situations, but are subject to horizontal–vertical anisotropy in others. Because the isotropy findings were a matter of results of experiments in open spaces, in which there was no visible border separating the space in chunks of spaces as in multi-level buildings, anisotropy might be primarily linked to the existence of such spatial compartmentalization. In fact, this fits the debate of environmental (e.g., multi-floor buildings) versus vista (or open) spaces, in which it has been shown that in general clustered, navigable spaces are represented qualitatively differently from open spaces because of the compartmentalization of space (Marchette et al. 2017; Meilinger et al. 2016). However, because the current evidence is indecisive regarding open spaces, in which potentially different processes occur compared with multi-floor spaces, further research is necessary to elucidate the picture of (an)isotropy in such open spaces. We aim to tackle this in the present study.

The previous studies tested spatial location representations, that is: “where is location A relative to location B?” We pursued a different route, utilizing a more basic spatial property that is independent of specific locations in space, namely, the representation of traveled distances. To our knowledge, this was not tested in the context of vertical space yet. Investigating traveled distances in the context of horizontal and vertical spatial representations can also be motivated by the bicoded-map hypothesis. This hypothesis predicts that anisotropies between horizontal and vertical axes in spatial representations occur already on a level of self-translation processing independent to specific spatial locations (Jeffery et al. 2013). Accordingly, accuracy in a traveled distance estimation task should be higher for movements along the horizontal compared with the vertical axis.

The traveled distance estimation paradigm leads participants on a straight path and lets them estimate or reproduce the traveled distance (e.g., Harris et al. 2000). It can be regarded as a sub-process of the often-used path integration task. The neuronal path integrator is commonly assumed to integrate self-motion signals over time to generate an estimate about, where someone has traveled relative to an origin (Etienne and Jeffery 2004; Loomis et al. 1999; Mittelstaedt and Mittelstaedt 1982). Studies using these paradigms varied the type of self-motion signals that were available to the participants. These signals can be grouped into visual (optic flow) and non-visual cues (efference copies and inertial signals) (Mittelstaedt and Mittelstaedt 1973). We limited the available cue in this study to inertial cues, i.e., horizontal and vertical accelerations.

Humans can detect inertial cues through their otoliths in the vestibular system in the inner ear (Mittelstaedt 1999)—and probably also through somatic graviceptors (Mittelstaedt 1996). The otoliths comprise the utricle and saccule, which respond to linear accelerations (Fernández and Goldberg 1976).

Theoretically, the perceived acceleration information can be integrated over time to obtain an estimate of position change or traveled distance relative to an origin. Indeed, a series of the previous studies demonstrated that blindfolded participants, led passively on a straight path, while sitting on a mobile chair can reproduce traveled distances based on inertial cues quite accurately from memory (Berthoz et al. 1995; Harris et al. 2000; Israël et al. 1997). We used a task similar to the ones used before (e.g., Harris et al. 2000; Israël et al. 1997) in the present study, with the novelty of adding vertical self-translations.

Using this traveled distance estimation paradigm, we tested different hypotheses about the nature of human representations of traveled distances along horizontal and vertical axes in the sagittal plane (i.e., we did not test for leftward and rightward translations). Closely related to the bicoded-map hypothesis, we tested the tenability of an anisotropy model, in which variations in spatial representations occur along the horizontal and vertical axes. The model postulates that horizontal translations show higher accuracy compared with the vertical translation axis. However, a potential anisotropy might not simply be reflected only in differences between these two dimensions. There might be variations in accuracy within a dimension. For instance, a difference might occur between up- and downward translations meaning that an anisotropy also occurs within the vertical dimension. Such a variation can be derived from results of a study showing different sensitives for up- and downward self-translations (Nesti et al. 2014a). In addition, variations might also be expected within the horizontal dimension, because findings showed discrepancies in anticipating visible targets during forward and backward linear displacements (Israël et al. 1993). We, therefore, evaluated several different models of representations of traveled distance: an isotropy model (no variation as a function of translation direction), an anisotropy model which predicts differences between horizontal and vertical distance estimates, and more refined anisotropy models, which predict different accuracy along the horizontal forward–backward or along the vertical upward–downward axes, or both (detailed explanation can be found in “Data analyses”).

Moreover, we aimed to investigate whether a potential anisotropy is body or earth-centered. Again, this question relates to the bicoded-map hypothesis of Jeffery et al. (2013). Our and Kaplan’s (2013) interpretation of the bicoded-map hypothesis leads to the prediction that an anisotropy pattern should be body-centered, with higher accuracy for translations along the body-centered horizontal than the vertical plane. In other words, the traveled distance information is encoded relative to the travelers’ own body. Alternatively, the anisotropy pattern might be independent of the body and higher accuracy might result for translations along the earth-centered horizontal axis, regardless of the travelers’ body orientation. This question of body versus earth-centered representation can also be phrased along the debate of whether the spatial information is encoded in an egocentric or in an allocentric reference frame (Klatzky 1998). Reference frames are defined by a reference direction. It was suggested that the constant force of gravity provides an ideal earth-centered reference direction (Barnett-Cowan and Bülthoff 2013), which might be used to encode traveled distance information. The previous studies already aimed at disentangling the role of body and earth-centered reference frames for other kinds of stimuli by introducing different body orientations with respect to gravity (e.g., Hinterecker et al., accepted; Karnath et al. 1998). To test whether body or earth-centered anisotropy holds true in traveled distance representations, we too will introduce different body orientations (upright and 30° backward-pitch orientation) during the distance estimation task.

In sum, the previous psychological studies showed different results concerning anisotropies in representations of horizontal and vertical spatial locations in open spaces. The present study aims to further elucidate how humans represent horizontal and vertical space by testing anisotropies on the level of traveled distances based on inertial self-translations. For this purpose, we conducted a traveled distance estimation experiment with translations along the sagittal plane covering horizontal and vertical movement components and tested accuracy of traveled distances. We also tested whether an anisotropy pattern is body or earth-centered.

## Method

### Participants

Twenty-four healthy naïve subjects (8 females), aged 20–60 years (M = 28.62, SD = 8.14), were recruited. They gave written consent after oral and written instruction and confirmed that they were free from any known vestibular, neurological, cardiac, or spinal illnesses. If entitled, participants received monetary compensation. The Ethics Committee of the University Clinic of Tübingen approved this study (315/2016B01).

### Materials

#### Task

**Fig. 1:**
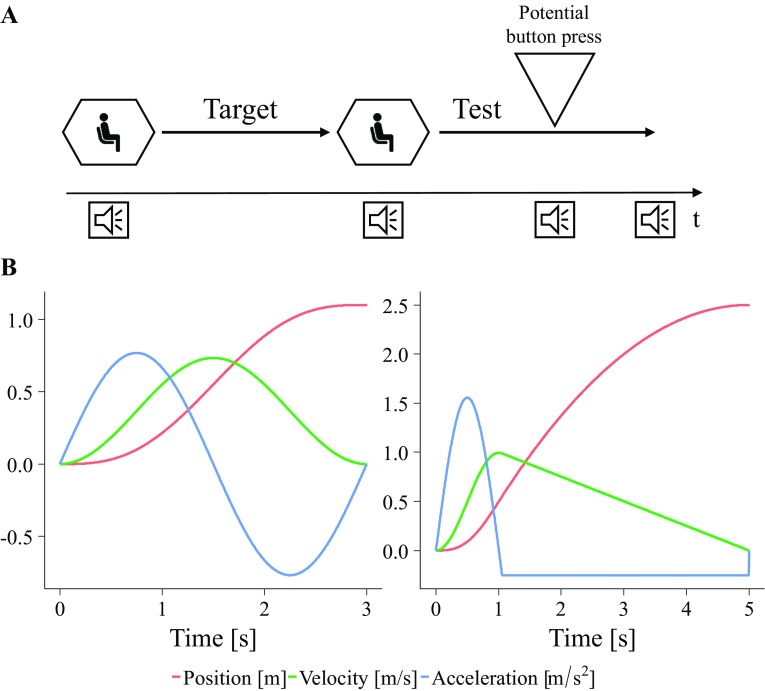
**a** Traveled distance estimation task was used in this experiment. Participants perceived two consecutive translational movements (target and test). The task was to indicate with a button press during the test translation when the participants perceived themselves to have traveled the same distance of the target translation. Participants heard sound beeps indicating the start of the target and test translation as well as the button press and the end of the trial. **b** Motion profiles for the target and test translations of Experiment 1. The left plot shows an example profile for a target distance of 1.1 m. The right plot shows the profile that was used for all the test translations

Participants performed a traveled distance estimation task (Fig. 1a). In this, they perceived two consecutive translational movements without active control while being blindfolded. The first translation was regarded as the target translation, the second translation as the test translation. The test translation differed from the target translation in its acceleration profile (Fig. 1b). Participants were required to memorize the traveled distance of the target translation and to press a button during the test translation as soon as they perceived themselves to have traveled the same distance of the target translation.

#### Stimuli

**Fig. 2:**
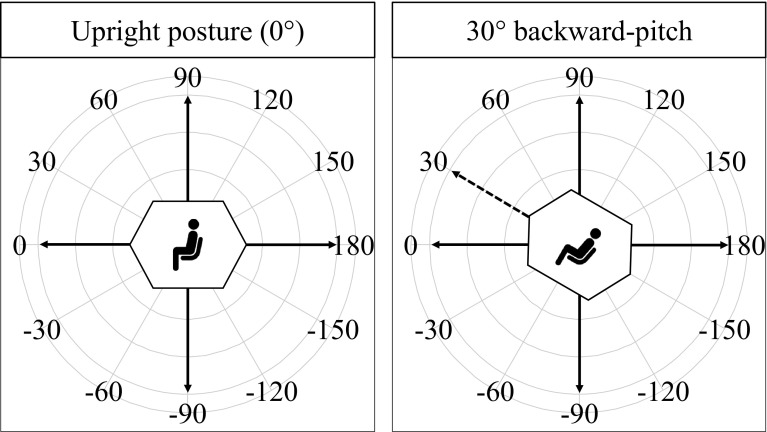
Experiment used two body orientations: upright and 30° backward-pitch (depicted by the tilted cabin and the dashed arrow in the right panel). In each condition, we used 12 different translation directions in the sagittal plane, varying from 0° (forward translation) to ± 180°, in ± 30° steps, as depicted at the periphery of the circle. The solid arrows represent the  earth-centered coordinate system. Participants carried out all trials for one body orientation condition first, before moving to the respective other condition

We used 12 translation directions along the participant’s sagittal plane (Fig. 2). The variation in translation direction ranged from 0° (straight forward) to ± 180°, in ± 30° steps. Hence, the amount of horizontal and vertical motion components differed across the 12 translation directions. Six target distances were used ranging from 0.5 to 1.5 m, in 0.2-m steps. Participants judged distances in each trial while being in an upright or in a 30° backward-pitch posture. The combination of the 12 translation directions, six target distances, and two body orientations led to a total of 144 test trials for each subject. Within a single trial, translation direction and body orientation were held constant. Participants performed the trials in 12 blocks. Each block consisted of 12 trials covering each translation direction once. For each translation direction, we assigned the target distances pseudo-randomly across all blocks. The first half of the trial blocks (72 trials) was carried out in either an upright or 30° backward-pitch orientation, the other half in the other respective orientation.

The target translation always lasted 3 s, such that participants could not use a strategy in which they reproduced the duration of the translation. The profile of the target translation was a raised cosine for velocity leading to a sinusoidal acceleration curve (Fig. 1b, left plot). The peak accelerations of the target translations varied from 0.35 m/s^2^ (target distance of 0.5 m) to 1.05 m/s^2^ (target distance of 1.5 m). For the test translation, always, the same motion profile was used, which differed from the profile of the target translation. The test profile consisted of a first acceleration using the first half of a sinusoidal profile followed by a constant deceleration (Fig. 1b, right plot). The velocity ramped up and then slowly decreased until the motion came to a halt at 2.5 m after 5 s. A peak acceleration of 1.55 m/s^2^ occurred. The constant deceleration was − 0.251 m/s^2^. These accelerations were above detection thresholds for linear acceleration reported in the literature (see Table 2 in Nesti et al. 2014a). Anytime during the test, profile participants could press the button to indicate that they now have traveled the same distance as in the target sequence. The test profile did not stop after a button press. Between the target and test translation, a break of 5 s occurred. Before each trial, the starting position was adjusted within the simulator workspace to account for the total distance to be covered during the target and test translations. This pre-positioning lasted 7 s, followed by a 5-s pause without any movement. The motion profiles differed to have participants concentrate on the distances and prevent them from potentially applying motion profile matching strategies.

During a trial, participants heard white noise through the speakers in the helmet. They also heard sound beeps indicating the start of a trial or target translation, the start of the test translation, the button press, and the end of a trial. These beeps varied in pitch, with a decrease in pitch from the start to the end of a trial. With the button press or at the end of a trial, the white noise stopped.

In addition, participants were exposed to wind generated by the two fans. We adjusted the fans before the start of the experiment to have one fan blowing wind onto the hands of the participants, while the other fan targeted the participants’ torso and head. The purpose of the artificial wind was to eliminate any somatosensory cue caused by varying airstream for the different translation directions. The fans were turned off between blocks.

To bring the participants into the 30° backward-pitch orientation, they were rotated accordingly. The necessary rotation was carried out at the beginning of a block and participants remained in this position during the whole block. Afterward, participants were brought back in their horizontal state. The rotations took 4 s.

#### Apparatus

**Fig. 3:**
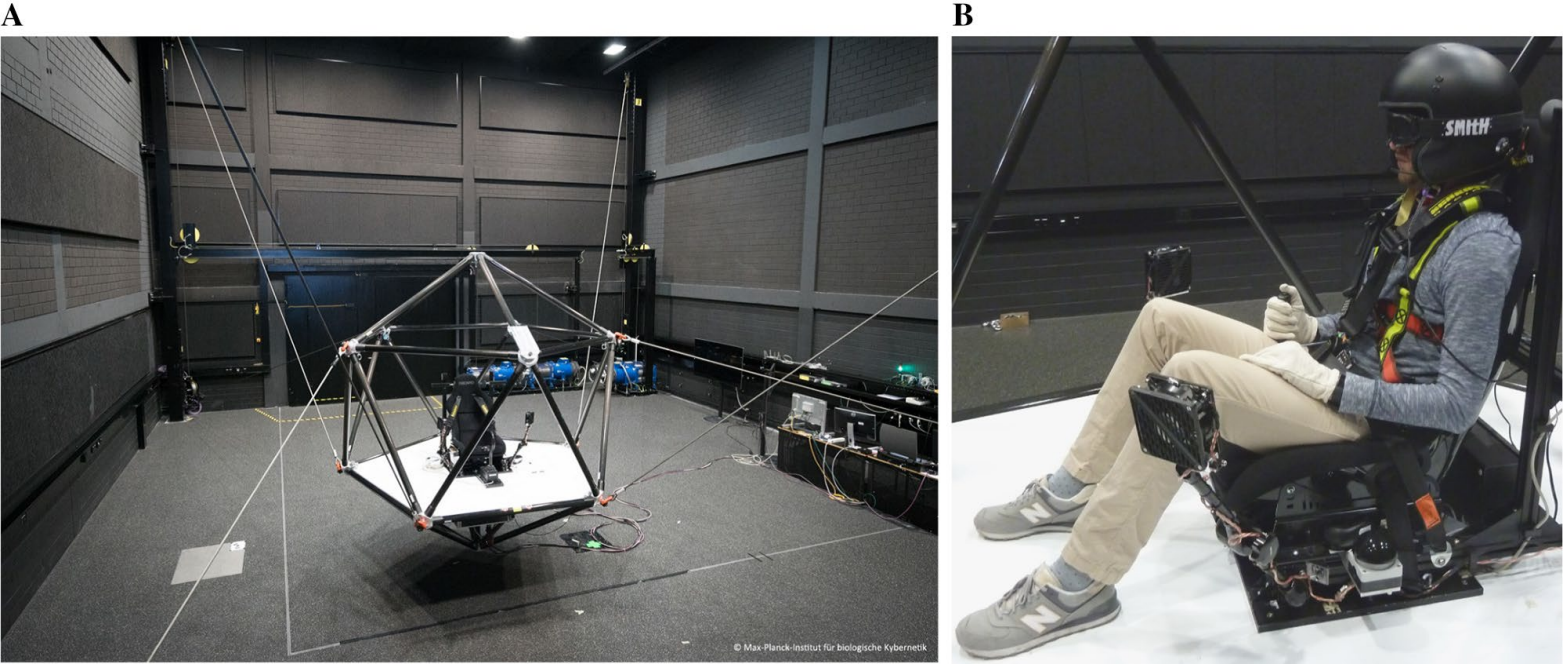
**a** MPI Cable Robot Simulator. **b** Participants were sitting in the seat mounted on top of the cabin platform. Participants were blindfolded and secured with seat belts. White noise played through the built-in speakers in the helmet masked auditory cues from the simulator, a taped ski-mask prevented visual motion cues. Fans mounted to the cabin were used to mask the airstream cues on hand, arm, and face caused by the motions. A HANS device protected participants from head and neck injuries. Participants held a button device, which they used to indicate traveled distances

The experiment was carried out using the MPI Cable Robot Simulator (Fig. 3a) (Miermeister et al. 2016). In this cable-driven simulator, electric motors control the extension of cables, pulling the cabin along freely programmable directions within a 4 × 8 × 4 m workspace. A racing chair with a five-point safety harness and an additional safety belt is attached to a horizontal surface.

Participant wore a helmet (Bell-Helm MAG-1 Rally, USA), and head and neck support device (HANS, HANS Performance Products, USA), which reduced the likelihood of head and/or neck injuries. It also limited head movements. The helmet has built-in speakers and a microphone enabling a continuous bi-directional communication between the participant and the experimenter. Participant wore a taped ski mask through which they could not see anything during the blocks of trials. Participants held a small button box in their preferred hand (Fig. 3b).

### Procedure

The first task for the participant was to read the safety instructions related to experiments using the MPI Cable Robot Simulator. Then, the instructor informed the participant about all relevant safety instructions verbally and questioned the participant on the exclusion criteria. If no exclusion criteria held, the participant read the detailed instructions of the experiment. Next, the instructor explained the traveled distance estimation task, the body orientation conditions, and the type of motions verbally and visually using a similar figure, as in Fig. 1a. Then, the participants watched a live demonstration of an exemplary simulator translation. This translation was straight upward from the lowest to the highest positions used in the experiment. The participant could ask any questions at any time.

Afterward, the participant put on the climbing harness, the HANS device, the Helmet, and the gloves, walked up a three-step staircase to the cabin and sat down on the racing chair. The experimenter made sure that the seat belts properly strapped the participant in. Then, the bi-directional communication devices were tested for proper function. Before the experiment started, the participant was moved straight upward to the highest position used in the experiment. This was done to familiarize the participant with the simulator motion before the experiment and to assure that the participant feels comfortable while being moved up to 5-m height. If everything was fine for the participant, the cabin was brought to the center of the simulator hall and the participant put the taped ski mask on.

The experiment started with four practice trials (two in the 30° backward-pitch body orientation). If the participant had any task-related issues during these trials (e.g., did not press the button or had problems with the sounds), the instructions were repeated briefly, and additional practice trials were granted until the participant understood the task properly. Afterward, the trials began in blocks of about 6 min. The participant performed the trials of the first six blocks either in the upright or 30° backward-pitch orientation. After each block, the participant could take a small break and take off the ski mask. After the first six blocks, a break of about 5–10 min occurred. During this break, the participant left the cabin and took off the helmet, etc. The experiment continued by carrying out the remaining six blocks of trials in the respective other body orientation. Participants were instructed to lean their heads to the back of the chair to have their head as upright as possible (the experimenter repeated this instruction throughout the experiment when necessary).

After conducting all trials on the motion simulator, participants filled out a questionnaire asking for strategies and self-evaluations. Afterward, the purpose of the study was revealed to the participant. Overall, the experiment lasted about 2 h.

### Design

The experiment consisted of a 2 × 12 × 6 within-factors design. The first factor, body orientation, consisted of two levels: an upright and a 30° backward-pitch orientation. The second factor, translation direction, consisted of 12 different directions in the sagittal plane encoded in an earth-centered coordinate system, ranging from 0° (straight forward) to ± 180°, in ± 30° steps (Fig. 2). The third factor, translations distance, consisted of six different distances.

### Data analyses

We subdivided the “Data analyses” section into three parts: (1) (an)isotropy model fit comparison; (2) analysis of body versus earth-centered anisotropy; and (3) stimulus noise analysis. All analyses were conducted using the software R (R Core Team 2017).

### (An)Isotropy model fits comparison

**Fig. 4:**
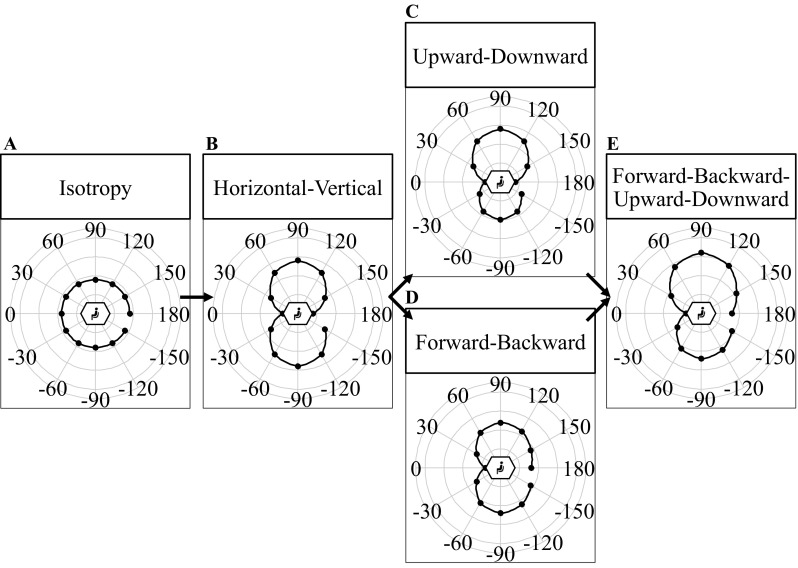
Panels show exemplary accuracy patterns for the isotropy model (**a**) and the four anisotropy models (**b**–**e**) tested in this study. The distance of the dots from the center of the plot represents the magnitude of estimation error. Smaller errors are shown for forward/backward translations, larger errors for upward and downward translations. The arrows represent the increase in the number of parameters and how the models are based on each other

We aimed to test (an)isotropy in the representation of traveled distances for different translation directions in the sagittal plane. To answer this question, we compared model fits. Regarding the anisotropy models, we not only tested for a horizontal–vertical anisotropy, but also tested for models with more detailed variations within the spatial axes (e.g., the difference between upward and downward). These were the so-called upward–downward anisotropy, the forward–backward anisotropy, and the forward–backward–upward–downward anisotropy model (Fig. 4).

We fitted a non-linear mixed effects models to the absolute error in the traveled distance estimation task and compared the goodness of fit of these models. The R package nlme was used for this purpose (Pinheiro et al. 2017). The mixed effects models used all the individual participants’ data points across all translation directions. The translation direction was treated as a continuous variable. The directions were used to calculate the horizontal and vertical components of the translation path utilizing the sine (vertical projection) and cosine (horizontal projection) functions. For instance, a translation direction of 90° resulted in a vertical translation component of one (sine of 90°), while the horizontal component was zero (cosine of 90°). In each model, these translation components were weighted with an individual free parameter. The resulting scalar value of a model was defined as the expected (or predicted) error in the distance estimation task. The mixed models allowed for random variability in the model parameters associated with the random effect of participants. The mathematical descriptions of the different anisotropy models are introduced in the following.

The horizontal–vertical anisotropy model consisted of a function with two parameters for weighting the absolute estimation error related to the horizontal and the vertical translation component, respectively. The mathematical description was

1 $${\text{Absolute~error}}\sim \sqrt {{{({w_{\text{h}}} \times \cos \alpha )}^2}+{{({w_{\text{v}}} \times \sin \alpha )}^2}} ,$$

where ${w_{\text{h}}}$ is the parameter for weighting the horizontal translation component, ${w_{\text{v}}}$ is the parameter for weighting the vertical translation component, and *α* is the heading angle in the sagittal plane. The weighted translation components were normalized (by taking the square root of the sum of the squared weighted translation components), because the model was required to result in the same value for the different translation directions, in the case of identical horizontal and vertical parameters.

The upward–downward anisotropy model refines the vertical component of the previous horizontal–vertical model, dividing it into two parameters for vertical translations. The model was defined as

2 $${\text{Absolute~error}}~\sim \sqrt {\begin{array}{*{20}{c}} {{{\left( {{w_{\text{h}}} \times \cos \alpha } \right)}^2}+} {\left( {\sin \alpha>0} \right) \times {{\left( {{w_{\text{u}}} \times \sin \alpha } \right)}^2}+\left( {\sin \alpha <0} \right) \times {{({w_{\text{d}}} \times \sin \alpha )}^2}} \end{array}} ,$$

where ${w_{\text{u}}}$ is the parameter for weighting an upward translation and ${w_{\text{d}}}$ is the parameter for weighting a downward translation. Note that the horizontal component ${w_{\text{h}}}$ is the same as above (model 1). Because a translation cannot be in an upward and downward (or forward and backward) direction at the same time, the respective term must be set to zero if the translation direction is to the opposite. This was implemented by the inequations (e.g.,$~\sin \alpha>0$). For instance, if the translation is downward, the sine of $\alpha$ is smaller than zero rendering the second term of Eq. 2 to zero, while the third term and, therefore, the upward weight are influencing the error value.

The forward–backward anisotropy model applies the same principle but introducing two parameters for horizontal translations. The model was defined as

3 $${\text{Absolute~error}}~\sim \sqrt {\left( {\cos \alpha>0} \right) \times {{\left( {{w_{\text{f}}} \times \cos \alpha } \right)}^2}+\left( {\cos \alpha <0} \right) \times {{\left( {{w_{\text{b}}} \times \cos \alpha } \right)}^2}+{{({w_{\text{v}}} \times \sin \alpha )}^2}} ,$$

where ${w_{\text{f}}}$ is the parameter for weighting a forward translation and ${w_{\text{b}}}$ is the parameter for weighting a backward translation. Note that the vertical component ${w_{\text{v}}}$ is the same as in model 1.

The forward–backward–upward–downward anisotropy model is a combination of the former two, and therefore, it uses all four direction-specific parameters introduced in models 2 and 3. It was defined as

4 $${\text{Absolute~error}}~\sim \sqrt {\begin{array}{*{20}{c}} {\left( {\cos \alpha>0} \right) \times {{\left( {{w_{\text{f}}} \times \cos \alpha } \right)}^2}+\left( {\cos \alpha <0} \right) \times {{\left( {{w_{\text{b}}} \times \cos \alpha } \right)}^2}} \\ + {\left( {\sin \alpha>0} \right) \times {{({w_{\text{u}}} \times \sin \alpha )}^2}+\left( {\sin \alpha <0} \right) \times {{({w_{\text{d}}} \times \sin \alpha )}^2}} \end{array}} .$$

The isotropy hypothesis was modeled using a linear mixed effects model including solely a random intercept for different participants. This model is a null or intercept model. Figure 4 shows predicted error patterns for the tested models.

The relative abilities of these models to describe absolute error in the traveled distance estimation task as a function of translation direction in the sagittal plane was quantified by the small-sample corrected version of the Akaike information criteria (AIC_c_) (Burnham and Anderson 2004). AIC_c_ provides a relative measure of the quality of the model. It assigns smaller values to models with a better trade-off between prediction accuracy and the number of coefficients.

### Body versus earth-centered reference frame

**Fig. 5:**
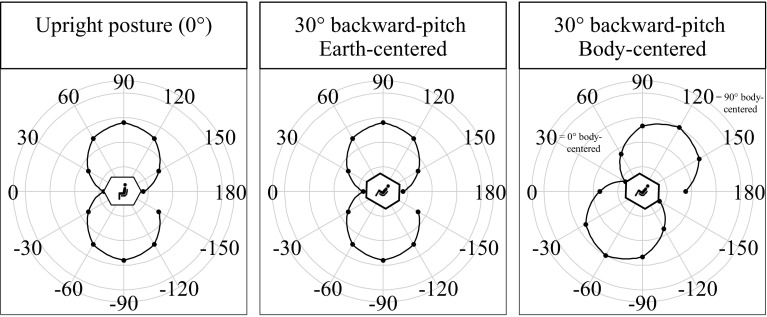
Example of body-centered (right panel) and earth-centered (middle panel) error patterns as a function of translation direction. In the left panel, in which the posture is upright, an exemplary horizontal–vertical anisotropy error pattern is shown. In the right panel, an exemplary body-centered pattern for the backward-pitch orientation is shown. The pattern is the same as in the upright condition but rotated by 30° (as the body). Hence, less error is shown for body-related forward translations. In contrast, in the middle panel, the pattern did not rotate with the body (the error pattern is earth-centered regardless of the body orientation), and therefore, less error is shown for the earth-centered forward translation

We aimed to test whether a possible anisotropy pattern is body or earth-centered (Fig. 5). For this purpose, the pattern of the upright condition was chosen as a reference and similarity with the pattern in the 30° backward-pitch condition was assessed. In the upright condition, both body and earth coordinates are aligned. For example, moving straight forward relative to the participant’s body corresponds to moving straight forward relative to the surrounding room (parallel to the floor) (Fig. 5, left). Whatever accuracy pattern that is shown by the participant might thus be body or earth-centered. The shift in body orientation in the 30° backward-pitch condition introduces a mismatch between the body and earth-centered reference systems and allows for a direct comparison with the reference pattern in the upright condition. If the anisotropy in the representation of traveled distances is body-centered, the pattern should rotate in accordance with the change in body orientation. In contrast, if the pattern is earth-centered, the pattern should not change. We assess this by coding the accuracy pattern of the 30° backward-pitch condition in two ways, either in a body-centered way (Fig. 5, middle)—e.g., 90° upward movement is defined relative to the body, corresponding to the accuracy pattern shown at orientation 120° in Fig. 5, middle—or earth-centered—e.g., 90° upward is defined relative to gravity, corresponding to the accuracy pattern shown at orientation 90° in Fig. 5, right. Those two versions of coding the accuracy in the backward-pitch condition were correlated with the accuracy in the upright condition. A significant correlation of the upright orientation pattern with the pattern of the backward-pitch orientation encoded in a body-centered coordinate system would indicate that the traveled distance information was encoded in a body-centered and not in an earth-centered reference frame, and vice versa.

### Stimulus noise analysis

In studies using motion simulators, uncontrolled simulator-induced noise can lead to disparity between the actual simulator motion and the desired stimulus. Such disparity can lead to undesired motion cues, which might affect the interpretation of experimental results (Nesti et al. 2014b). Following the recommendation and guidelines of Nesti et al., we conducted a stimulus noise analysis for the used trial motions in the Cable Robot simulator to test for simulator-induced noise patterns that might explain participants’ accuracy results in the traveled distance estimation task. For this purpose, the cabin motions for all trials were recorded via an inertial measurement unit (LandMark^™^ 01 IMU, Gladiator Technologies, USA) attached to the simulator cabin. The recordings were repeated five times for each trial. Before analyzing the data, we applied a low-pass filter (removing frequency components above 80 Hz) and removed the gravity signal from the recordings (by setting the mean intercept of the recorded signals to zero). Then, we subtracted the intended acceleration signal from the acquired IMU signal to obtain the stimulus noise. Afterward, we calculated the signal-to-noise ratios (SNR) for each of the possible target and test translations. Finally, we analyzed whether these SNRs varied significantly across translation directions and whether they can explain our psychophysical results by correlating the SNR pattern with the accuracy pattern. The equation used for calculating the SNRs using the commanded and recorded signals is the following:

5 $${\text{SNR}}={\left( {\frac{{{\text{rms}}\left( {\sqrt {{\text{cm}}{{\text{d}}_x}^{2}+{\text{cm}}{{\text{d}}_y}^{2}+{\text{cm}}{{\text{d}}_z}^{2}} } \right)}}{{{\text{rms}}\left( {\sqrt {{{\left( {{\text{cm}}{{\text{d}}_x} - {\text{re}}{{\text{c}}_x}} \right)}^2}+{{\left( {{\text{cm}}{{\text{d}}_y} - {\text{re}}{{\text{c}}_y}} \right)}^2}+{{\left( {{\text{cm}}{{\text{d}}_z} - {\text{re}}{{\text{c}}_z}} \right)}^2}} } \right)}}} \right)^2},$$

where rms is the root mean square, cmd stands for commanded motion and rec for recorded motion.

## Results

### (An)Isotropy model fits comparison

Four participants did not obtain a significant correlation between the actual distances and the estimated distances, which indicates that they were not able to replicate the traveled distances. They rather pressed the button repeatedly at the same time during the test translation, by potentially applying a duration estimation strategy (although the instructions made clear that such a strategy is not appropriate for solving the task). We excluded them from the following analyses. For the remaining data, we excluded all trials in which the absolute error deviated more than two standard deviations from a participants’ overall mean (4.1% of all trials).

**Fig. 6:**
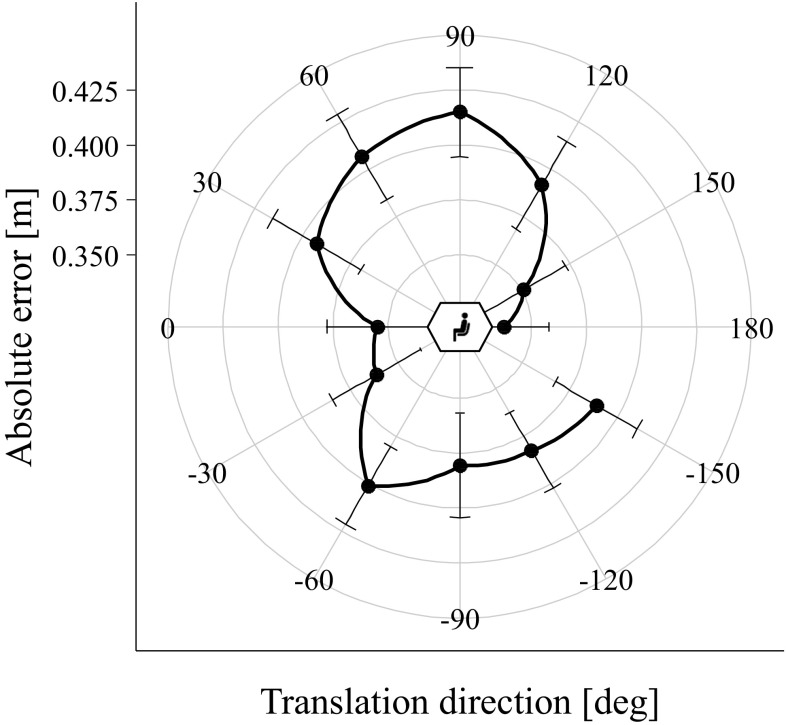
Absolute error (in meter) in the traveled distance estimation task as a function of translation direction in the sagittal plane (12 directions between − 150° and 180° meter in 30° steps). Zero degrees was a forward translation, 90° an upward translation. Error bars display standard errors of the mean

Figure 6 indicates variation in absolute error as a function of translation direction. To test whether there is statistical evidence for anisotropy in representations of traveled distance, the isotropy and the four anisotropy models (formulas 1–4 in “Data analyses”) were fitted to the participants’ data of the upright conditions. The model with the lowest AIC_c_ (better) was the horizontal–vertical anisotropy model (see Table 1 for dAIC_c_ values). The horizontal–vertical anisotropy models’ parameter reflecting the associated absolute error for translations along the horizontal axis was 0.36; the parameter for translations along the vertical axis was 0.40. This indicates higher accuracy for translations along the horizontal compared with the vertical axis.

**Table 1:** dAIC_c_ values for the fitted iso- and anisotropy models

Model	Body orientation		
	Upright posture	30° backward-pitch	
		Earth-centered	Body-centered
Isotropy	5.6 (− 57.9)	0 (− 52.5)	0 (− 52.5)
Horizontal–vertical	0 (− 63.5)	3.2 (− 49.3)	1.4 (− 51.1)
Upward–downward	4.6 (− 58.9)	6.5 (− 46.0)	7.8 (− 44.7)
Forward–backward	7.4 (− 56.1)	9.2 (− 43.3)	8.2 (− 44.3)
Forward–backward–upward–downward	14.2 (− 49.3)	12.7 (− 39.8)	16.6 (− 35.9)

For each body orientation condition (upright orientation, earth-centered and body-centered 30° backward-pitch orientation), the model with the lowest AIC_c_ value is highlighted (best fit) and taken as the reference for comparison with the other models, thereby set to zero. For all remaining models, the difference in AIC_c_ value to the best fitting model is shown (dAIC_c_). The corresponding AIC_c_ values are shown in parentheses

### Body versus earth-centered reference frame

**Fig. 7:**
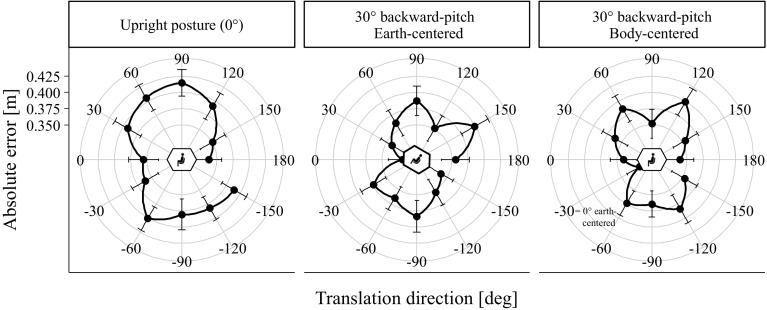
Absolute error (in meter) in the traveled distance estimation task as a function of body orientation (upright and backward-pitch) and translation direction in the sagittal plane. The left plot shows the same data as presented in Fig. 6. The data for the 30° backward-pitch condition are displayed twice. In the middle plot, it is shown in an earth-centered coordinate system. In the right plot, the same data are presented in a body-centered reference frame (the data points were simply rotated counterclockwise by − 30°). Both types of encoding of the data acquired in the backward-pitch condition were correlated with the data of the upright condition to test for a body or earth-centered anisotropy

To test whether the anisotropy in traveled distances was body or earth-centered, we correlated the obtained absolute error pattern across the translation directions of the upright orientation with the accuracy pattern of the 30° backward-pitch orientation for both earth- and body-centered coordinates (Fig. 7). We observed a significant correlation with the body-centered pattern, *r*(12) = 0.58, *p* = 0.049, but not the earth-centered pattern, *r*(12) = 0.07, *p* = 0.840. These results suggest that the pattern of anisotropy was body-centered.

### Stimulus noise analysis

**Fig. 8:**
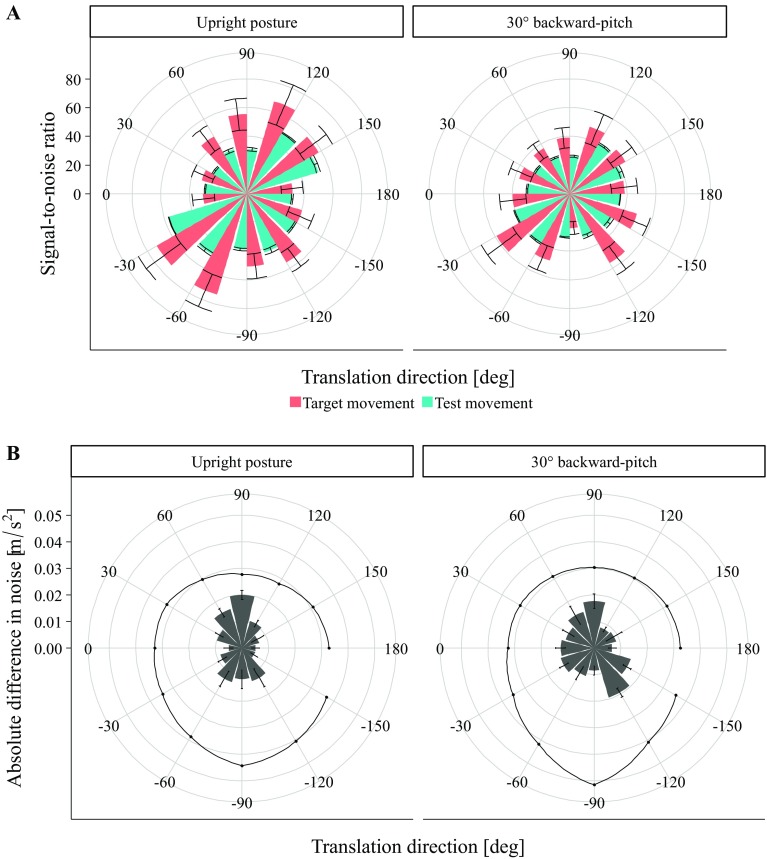
**a** Signal-to-noise ratios for the target and test translations of the experimental trials for both body orientations as a function of translation direction. **b** Absolute difference in RMS of the stimulus noise between the target and test translations across translation directions. The solid line indicates the lowest calculated differential threshold for accelerations in the current literature across the translation directions (obtained from Naseri and Grant 2012; Nesti et al. 2014a)

The obtained SNRs for the target and test motions as a function of translation direction are presented in Fig. 8a. We calculated ANOVAs with the factors translation direction and body orientation to test for significant effects of these factors. For both the target and test translations, the ANOVAs revealed significant main effects and interactions (*F*s > 5.10, *p*s < 0.001). This indicates that SNR varied significantly as a function of translation direction, of body orientation, and the combination of both. To test whether these variations in SNR might pose an alternative explanation for the results of our experiment, we correlated the SNRs for the target and test translations with the participants’ accuracy patterns for both body orientations. No significant correlations were obtained (*r* < 0.28, *p* > 0.393). This indicates that these SNR patterns alone cannot explain the variations in participants’ results across the translation directions.

As can be seen in Fig. 8a, the SNR for the test translations (blue bars) was numerically smaller compared with the SNR of the target translations (except for the straight downward translation in the 30° backward-pitch body orientation). This observation led to a post-hoc hypothesis stating that reproduction accuracy in the distance estimation task is influenced when the noise level of the test translation is of larger magnitude than of the target translation. Possibly, if there is more noise during the test as compared with the target translation, accuracy might be reduced, because the information that is available to compute the distance is less reliable. Furthermore, if such differences in SNR between the target and test translations vary significantly across translation direction, they might pose an explanation for our psychophysical results. To this end, we subtracted the SNR of the test translation from those of the target translation and performed an ANOVA to test for significant variations in this difference in SNR across translation directions and body orientation. The ANOVA did not reveal any significant effects (*F*s < 0.80). This suggests that the magnitude of difference in SNR between the target and test translations did not vary much as a function of direction. Nevertheless, we correlated the differences in SNRs with the results for both body orientations and obtained a significant correlation for the upright posture, *r*(10) = 0.67, *p* = 0.017, but not for the 30° backward-pitch orientation, *r*(10) = − 0.11, *p* < 0.740. This suggests that accuracy in the traveled distance estimation task in the upright condition decreased with an increase in the difference in signal-to-noise ratio between the target and test translations.

As a next step, we estimated whether the differences in simulator noise levels between the target and test translations were in a range perceivable for humans (see Fig. 8b) and consulted perceptual thresholds reported in the literature. In general, two different kinds of perceptual thresholds are reported: absolute and differential thresholds. Absolute thresholds describe the smallest level of stimulus intensity (here acceleration) that is detectable. Differential thresholds describe the smallest detectable difference in stimulus intensity between two stimuli. As participants related two stimuli in our experiment, we referred to differential thresholds for testing whether the differences in noise level between our target and test translations were perceivable. We obtained differential thresholds for every translation direction by calculating threshold values for every possible stimulus (different combinations of direction and distance) in our experiment using differential threshold functions reported in the literature (Naseri and Grant 2012; Nesti et al. 2014a). For horizontal translations, the function reported by Naseri and Grant (2012) that led to smaller overall thresholds values was used:

6 $$\Delta I=0.05 \times I+0.03,$$

where $\Delta I$ is the difference in stimulus intensity required to be perceived, and *I* stands for stimulus intensity.

For vertical translations, two different functions reported by Nesti et al. (2014a, b) were used. These were separate functions for upward:

7 $$\Delta I=0.19 \times {I^{0.60}}$$

and downward translations:

8 $$\Delta I=0.17 \times {I^{0.42}}.$$

The stimulus intensities that were plugged into these formulas were the root mean square of the noise of the target motion. Because some translation directions were along both the horizontal and vertical axes, differential threshold values had to be calculated using Eq. 6 together with Eqs. 7 or 8. For this purpose, the following equation was used:

9 $$\Delta I=~\sqrt {\begin{array}{*{20}{l}} {{{(\cos \left( \alpha \right) \times 0.05 \times I+0.03)}^2}} \\ +{\left( {\sin \left( \alpha \right)>0} \right) \times {{(\sin \left( \alpha \right) \times 0.19*{I^{0.60}})}^2}} \\ +{\left( {\sin \left( \alpha \right)<0} \right) \times {{(\sin \left( \alpha \right) \times 0.17*{I^{0.42}})}^2}} \end{array}} ,$$

where *α* stands for the angle of translation direction in the sagittal plane. If the translation was in an upward direction, the threshold for downward translations was set to zero, and vice versa.

As can be seen in Fig. 8b, the lowest differential thresholds obtained by this procedure (indicated by the solid line) are consistently higher than the differences in noise between the target and test translations across the translation directions. When calculating an ANOVA to test whether the differential threshold values were significantly different from the difference in simulator noise, we obtained clear support for that (*F*s > 127.00, *p*s < 0.001). This indicates that differences in simulator noise between the target and test translations were not large enough to be perceived by the participants. The significant correlation of the SNR pattern in the upright posture with the participants’ accuracy data might, therefore, be irrelevant. Together with the results of the ANOVA testing significant variations in the difference in SNR across translation directions and body orientation these findings make it rather unlikely that the differences in signal quality between the target and test translations influenced participants’ accuracy.

## Discussion

We investigated whether human representations of horizontal and vertical traveled distances perceived by inertial self-motion cues are subject to iso- or anisotropy (equal versus different accuracy along the spatial axis). Our results indicate a horizontal–vertical anisotropy in traveled distance representations, with higher accuracy for translations along the horizontal compared with the vertical axis. We also tested whether this anisotropy is body or earth-centered. Correlations between the accuracy patterns of the upright and the backward-pitch orientation encoded in a body-relative coordinate system are suggestive of a body-centered anisotropy. These findings extend the previous psychological results regarding anisotropies.

Although studies report higher accuracy in memory for horizontal than vertical spatial locations in multi-floor buildings (Büchner et al. 2007; Hölscher et al. 2006; Montello and Pick 1993; Thibault et al. 2013; Zwergal et al. 2016), evidence regarding such anisotropies in open spaces has been indecisive. Our results indicate an advantage in human representations for spatial information along the (egocentric) horizontal over the vertical axis in open spaces. This horizontal advantage is in line with the findings in multi-floor buildings. However, studies on memory for spatial locations in open spaces reported isotropic representations (Hinterecker et al. 2018; Kim et al. 2017). How could this discrepancy be explained? Because our and these studies used different methodologies, different factors might contribute to whether spatial representations are subject to isotropy or horizontal–vertical anisotropy in open spaces.

First, neither Hinterecker et al. (2018) nor Kim et al. (2017) had participants encode the spatial information via real physical self-movement. Hinterecker et al. (2018), had participants learn a spatial layout visually and without any locomotion. Kim and colleagues used visual motion in a virtual reality setup. Both found isotropy in horizontal and vertical spatial representations. In contrast, real physical self-motion was used in the present and in the study of Barnett-Cowan et al. (2012), both showing performance patterns supporting anisotropic spatial representations. This renders it possible that anisotropies in open spaces are only found with real physical self-translations.

Second, learning a regular lattice structure (Kim et al. 2017) or a grid of target objects (Hinterecker et al. 2018) might allow for categorical encoding (Huttenlocher et al. 1991). Specifically, participants in both of these studies might have encoded spatial information categorically along levels, rows, and columns. In contrast, the present and the study of Barnett-Cowan et al. (2012) did not provide categorical cues to structure the traveled space. In both studies, participants had to rely on isometric encoding such as “1.1 m forward.” Thus, horizontal–vertical anisotropy in open spaces might depend on categorical versus isometric encoding of spatial information, with categorical encoding not leading to anisotropy.

Besides the horizontal–vertical anisotropy model, we tested for other, more detailed anisotropy models (Fig. 4c–e), because the previous studies indicate discrepancies in the accuracy of traveled distance estimations in forward versus backward translations (Israël et al. 1993) or different sensitivities for upward versus downward translations (Nesti et al. 2014a). However, these models did not fit to the data better compared with the horizontal–vertical anisotropy model. Even though strongest support was found for the horizontal–vertical model, we are hesitant to interpret our findings as evidence against the existence of more detailed variations of anisotropy in spatial memory. Forward–backward or upward–downward anisotropies might exist, but could not be captured with our experiment and analysis. One reason for the shortcoming of the more detailed models might be related to characteristics of the AIC_c_, which was used to select the best fitting model. The AIC_c_ values the best trade-off between prediction accuracy and the number of parameters. The observed differences in overall accuracy across translation directions in terms of accuracy might have been too small to lead to a better AIC_c_-based trade-off for the models that possess a higher number of parameters. More sensitive measurements of traveled distance might identify more fine-grained anisotropies. One possibility to achieve this might be linked to longer distances, for instance. Nonetheless, the primary message concerning the question of isotropy versus anisotropy in representations of horizontal and vertical traveled distances remains.

Another question of our study was whether the anisotropy and, therefore, the representation of traveled distance based on inertial self-translation was centered on the body or the earth coordinate axes defined by gravity. This closely relates to the debate of whether spatial information is encoded in an egocentric or in an allocentric reference frame. Our finding suggests a body-centered anisotropy, because the accuracy patterns of the upright condition were positively correlated with the body-relative (not the earth-relative) coordinate mapping of the backward-pitch condition. It suggests that traveled distance information based on inertial self-translations is not transformed in an allocentric earth-centered reference frame. This finding is in line with other studies investigating the role of an allocentric reference frame defined by gravity, which reported egocentric patterns too (Hinterecker, accepted; Karnath et al. 1998; MacNeilage et al. 2010). Overall, our findings concur with the bicoded-map hypothesis, which predicts higher accuracy for spatial information along egocentric horizontal than vertical axis (Jeffery et al. 2013).

Whereas we used the accuracy data of the upright condition to determine which of the (an)isotropy models holds true, we also examined the best fitting model in the 30° backward-pitch condition encoded in an earth-centered and a body-centered reference frame (Table 1). For both types of coding, the isotropy model had the lowest AIC_c_ value. However, according to the model selection guidelines of Burnham and Anderson (2004), the difference between the AIC_c_ values of the isotropy model and the horizontal–vertical anisotropy model is not large enough to distinguish these two models (difference smaller than 4). Thus, no clear decision regarding which model explains the data of the backward-pitch condition better can be made. However, the anisotropy results of the upright condition as well as the correlation of the (body-centered) accuracy patterns of both conditions render the horizontal–vertical model altogether more likely. Noteworthy, comparing the AIC_c_ values of the earth-centered and body-centered data of the backward-pitch condition reveals a lower value for the horizontal–vertical model in the body-centered coordinate mapping. This is consistent with the obtained correlation suggestive of a body-centered anisotropy.

The task of this study required the integration of the perceived acceleration signal of the self-translations. This integration process is part of path integration (Etienne and Jeffery 2004; Loomis et al. 1999; Mittelstaedt and Mittelstaedt 1982). The study of Barnett-Cowan et al. (2012), which used a point-to-origin task, indicates anisotropic human path integration. Such anisotropies might arise in different brain areas linked to path integration (Indovina et al. 2016). Our study refines these findings by presenting results indicating that its distance estimation sub-process is subject to horizontal–vertical anisotropy. What might be the cause of this? Path integration can generally be seen as a process that relies on working memory and possesses a limited amount of capacity (Waller and Greenauer 2013; Wan et al. 2013). The ability of the working memory system to represent and maintain the relevant information for the traveled distance integration process might vary as a function of horizontal and vertical translation directions. This might simply be related to the fact that humans travel more frequently along the egocentric horizontal than vertical axis (Barnett-Cowan et al. 2012).

Besides, neurophysiological explanations might exist for the observed anisotropy. A recent study revealed a direct connection between path integration and grid cells in mice (Gil et al. 2018). This suggests that a potential horizontal–vertical anisotropy in path integration is because of anisotropic grid cell activity (Hayman et al. 2011). The previous studies provided evidence for the existence of grid cells in humans (e.g., Doeller et al. 2010; Jacobs et al. 2013). It has been reported that these cells also function in darkness (Hafting et al. 2005), which suggests inputs of inertial cues. Accordingly, the here-observed anisotropy based on inertial self-motion cues could be attributed to such grid cell activity in humans too. However, as long as the grid cell functionality for vertical compared with horizontal self-translations in humans is unknown, this remains speculation.

Instead, the observed anisotropy might be attributed to discrepancies in physiological processes responsible for the perception of inertial self-translation cues. One possibility is that it arises at the sensory level. Specifically, the anisotropy might be caused by the biological make-up of the otoliths. Fernández and Goldberg (1976) found anisotropies in the behavior of sensory neurons of the otoliths to linear accelerations in the squirrel monkey. Their results show a non-uniform distribution of preferred heading directions and predictions based on these findings were confirmed in studies on heading perception in the horizontal plane (front–back–left–right) in humans (Cuturi and MacNeilage 2013; de Winkel et al. 2015, 2017). Fernández and Goldberg (1976) also showed 30% higher otolith-based sensitivity for egocentric horizontal than vertical translations, which is reflected in direction discrimination thresholds in humans (Benson et al. 1986; MacNeilage et al. 2010). These results indicate that the horizontal–vertical anisotropy in traveled distance representation of the present study can be a product of varying accuracy in otolith afferent neurons. It suggests that already the sensory information is subject to a horizontal–vertical anisotropy. The integration process responsible for computing the traveled distance might then simply suffer from the forwarded anisotropic linear acceleration signal.

However, more recent findings reported a similar sensitivity of otolith afferents of macaque monkeys when responding to horizontal or vertical linear acceleration (Jamali et al. 2009; Yu et al. 2012). These findings challenge conclusions from the results of Fernández and Goldberg (1976) and it remains unclear whether the human vestibular system is more sensitive to horizontal over vertical motion. Hence, it is possible that the results in humans concerning horizontal and vertical self-translations are not a reflection of anisotropies in the vestibular system but of later perceptual processing stages of the sensory information. Potentially, anisotropies might arise in different vestibular-responding areas in the brain (Yu et al. 2012). One candidate area might be the dorsal medial superior temporal area, where neurons have been found to show non-uniform distributions of preferred heading directions in monkeys too (Gu et al. 2010).

In sum, it is likely that the observed anisotropy in traveled distance representation based on inertial self-motion cues is caused by an anisotropic perception of these cues. The integration process for computing traveled distances might then show anisotropies as a result of this. This does not exclude the possibility of human grid cell activity showing such anisotropies as a response to inertial self-translations. However, such grid cell response might then also simply be attributed to earlier anisotropic perceptual processes. More research is required to clarify this and the question of at what stage the observed anisotropy in human traveled distance representations emerges.

Independent of this, the observed horizontal–vertical anisotropy is consistent with previously reported findings on human differential thresholds regarding inertial self-translations. Indeed, differential thresholds for linear accelerations in the horizontal plane (Naseri and Grant 2012) have been reported to be lower when compared with results regarding the vertical plane (Nesti et al. 2014a). Predictions based on these findings lead to a horizontal–vertical anisotropy for tasks involving discrimination of inertial self-translations. Our experiment confirms this prediction, with the novelty of testing for both horizontal and vertical motions in a single setup and of abstracting from a purely perceptual-based (discrimination of accelerations) to a more memory-based task (reproduction of traveled distance based on path integration).

Can the simulator noise explain our accuracy of distance estimation? The conducted analysis revealed absolute SNR patterns that did not correlate with the findings of our experiment. This suggests that there is no such connection between simulator noise and participants’ accuracy. However, when correlating the differences in SNRs between the target and the test translations of our estimation task, a substantial correlation with the accuracy results in the upright orientation occurred. A higher difference in SNRs was associated with a higher error in our distance estimation task. In other words, greater differences in noise level between the target and the test translation led to greater estimation error. On first sight, this correlation appears troublesome, as it poses an alternative explanation to any cognitive or physiological cause we hypothesize for the anisotropy of horizontal and vertical traveled distances. Yet, we do not think that it can explain our data. First, the differences in SNRs did not differ significantly across the translation directions. This means that the correlation with the accuracy data might be irrelevant at first place. Second, the patterns for the backward-pitch orientation did not correlate. If there is a connection between the simulator noise and the behavioral measures, then this should become evident in all conditions. Third, comparing the difference in noise level to perceptual differential thresholds that we calculated using formulas reported in the current literature (Naseri and Grant 2012; Nesti et al. 2014a) revealed that the variations in simulator noise level were probably not perceivable for participants. Therefore, it seems rather unlikely that the variations in participants’ accuracy stem from the differences in simulator noise.

A potential concern regarding the setup of our study is related to the used body posture. Because of characteristics of the simulator setup (Fig. 3), the legs of participants were slightly more bended compared with when sitting on a typical chair. This may have influenced participants because of a potential uncomfortable or unusual posture. However, participants did not complain about the body and leg posture. In addition, similar setups were used previously, in which the body posture was considered seated and not unusual (e.g., de Winkel et al. 2018; Nesti et al. 2017). Another concern is related to less accurate body orientation perception in a seated posture (Israël and Giannopulu 2012) when compared with situations with outstretched legs (e.g., as during normal standing or lying down with non-bended legs) (Cohen and Larson 1974). This finding might be related to graviceptors in the human trunk (Mittelstaedt 1996), with the seated posture leading to different body orientation estimates because of a shifted center of mass compared to a standing or lying posture. Because these somatic graviceptors probably also provide cues of linear movement (Mittelstaedt 1997), distance estimations, while standing or lying might as well lead to different results in terms of overall accuracy when compared with a seated posture. Providing certainties regarding the effects of different body postures on traveled distance estimations is beyond the scope of this study and future experimentation is required to investigate this question. However, when keeping the head posture upright regardless of a seated or non-seated body posture, we would predict similar anisotropy results, as the primary perception of the movements should happen in the vestibular system located in the head.

To conclude, our experiment was the first investigating human representations of non-visually perceived traveled distances for different horizontal and vertical self-translation. It showed that accuracy of these representations is subject to a horizontal–vertical anisotropy. Higher accuracy was associated with the horizontal axis, whereas an increase in the vertical translation component led to a decrease in accuracy. In addition, traveled distances seem to be encoded in a body-centered reference frame, in which the anisotropy relates to body-related translation directions. This finding is consistent with findings on motion perception. It suggests that the distance estimation sub-process of path integration is subject to horizontal–vertical anisotropy. It further adds to the diverse picture of human horizontal–vertical spatial representations in open spaces and highlights the need to investigate further the factors influencing the genesis of iso- or anisotropic patterns, such as real physical self-translations or categorical versus isometric encoding of space.
